# Supplementary figures and images for: Neural correlates of eye contact and social function in autism spectrum disorder
Source: PLoS One. 2022 Nov 9;17(11):e0265798. doi: 10.1371/journal.pone.0265798 (PMC9645655; doi:10.1371/journal.pone.0265798)

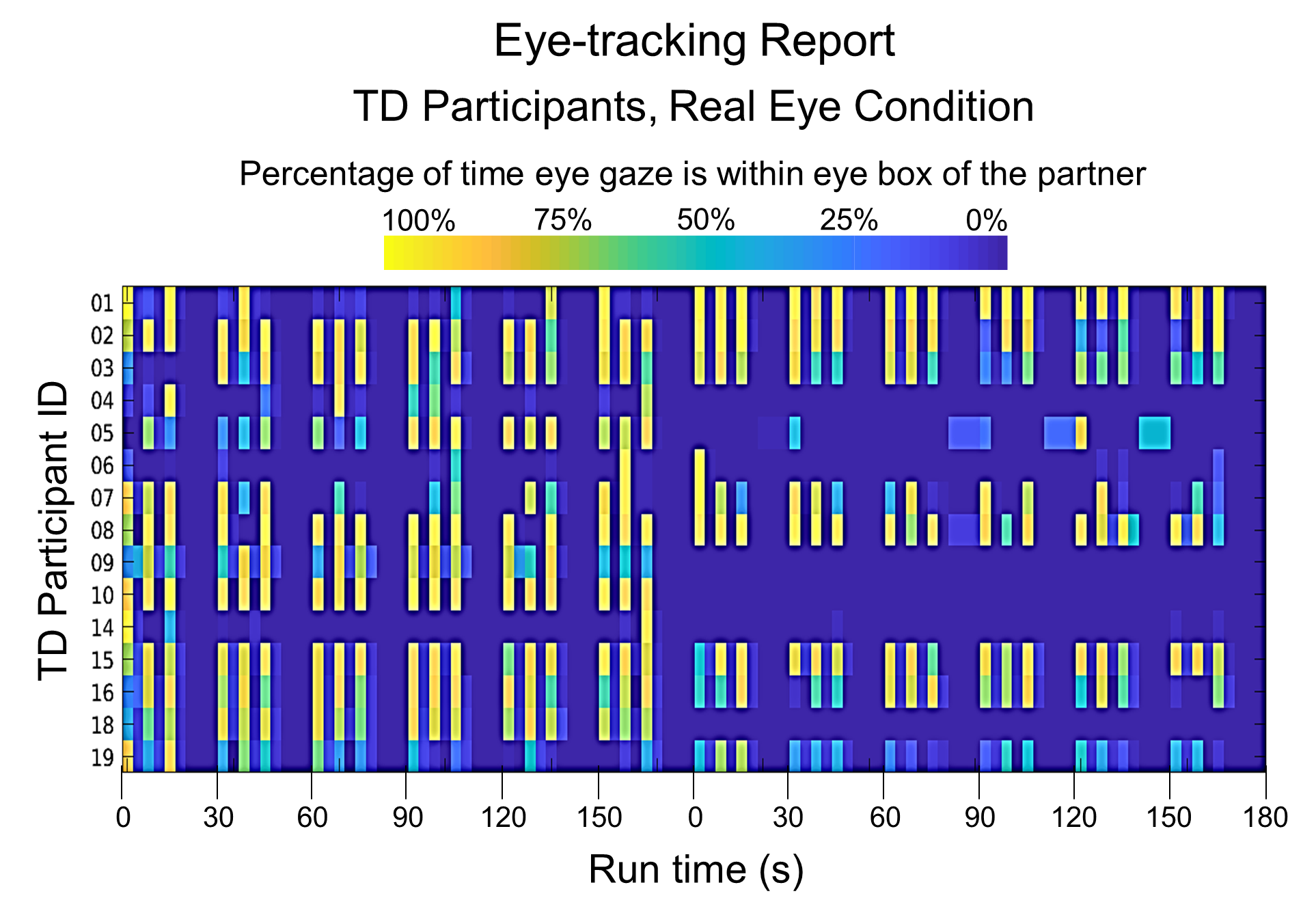

Supplement: S2 Fig — Colors indicate the percentage of time eye gaze is within the eye region of the partner (dark blue = 0% and bright yellow = 100%) during each epoch of the time series (x-axis). The vertical axis includes all TD participants for whom eye tracking data were acquired. (TIF) [file pone.0265798.s002.TIF]

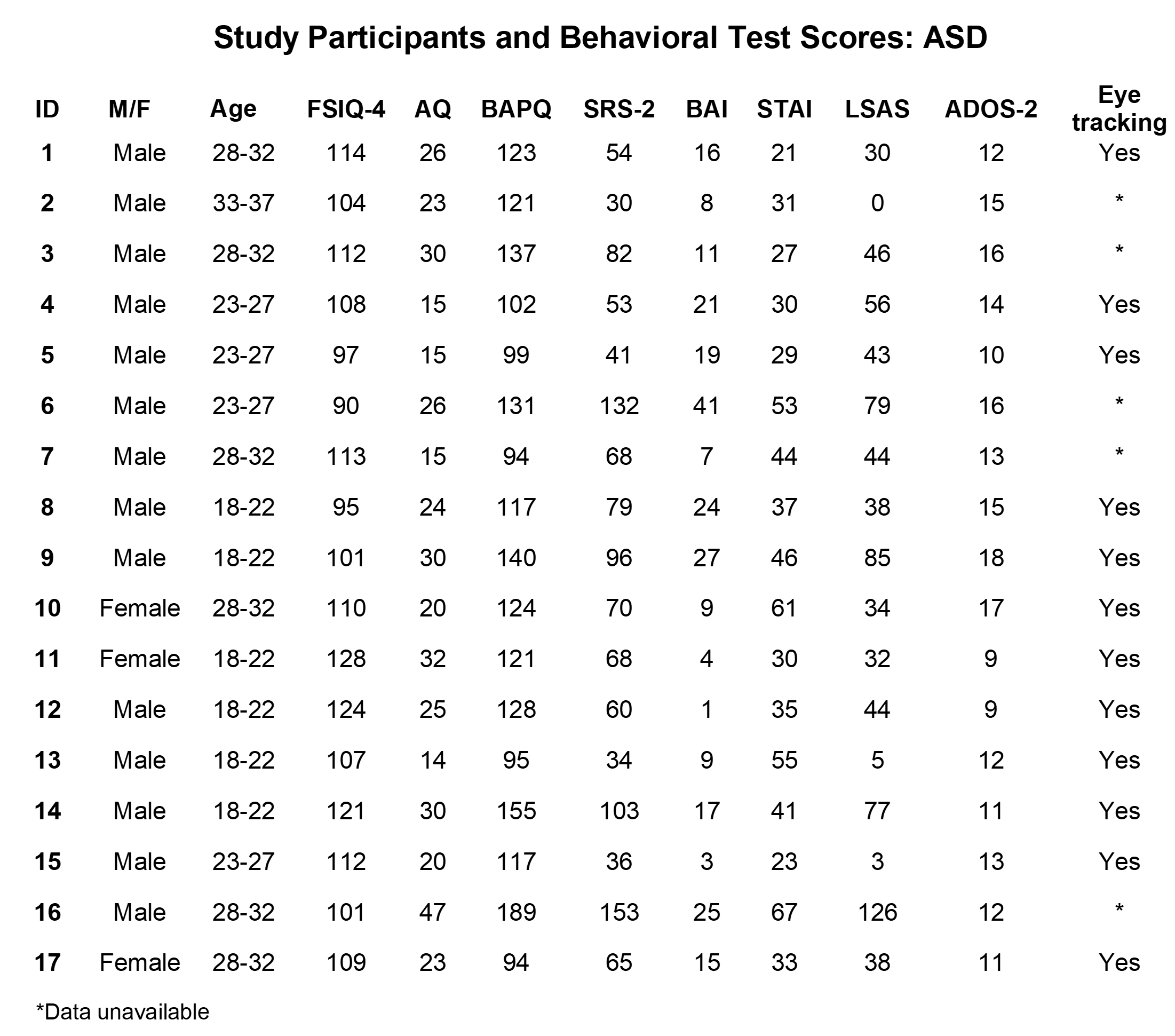

Supplement: S1 Table — Assessment measures include the Autism-Spectrum Quotient test (AQ, total scores); Broad Autism Phenotype Questionnaire (BAPQ, total scores); Social Responsiveness Scale, Second Edition (SRS-2, raw scores); Beck Anxiety Inventory (BAI, total scores); State-Trait Anxiety Inventory (STAI; total state anxiety scores); Liebowitz Social Anxiety Scale (LSAS, total scores); and the Autism Diagnostic Observation Schedule (ADOS-2, total scores). The Wechsler Abbreviated Scale of Intelligence, 2nd Edition (WASI-II) was administered to estimate full-scale intelligence quotient scores based on four subtests (FSIQ-4). *Indicates data were not acquired. (TIF) [file pone.0265798.s003.TIF]

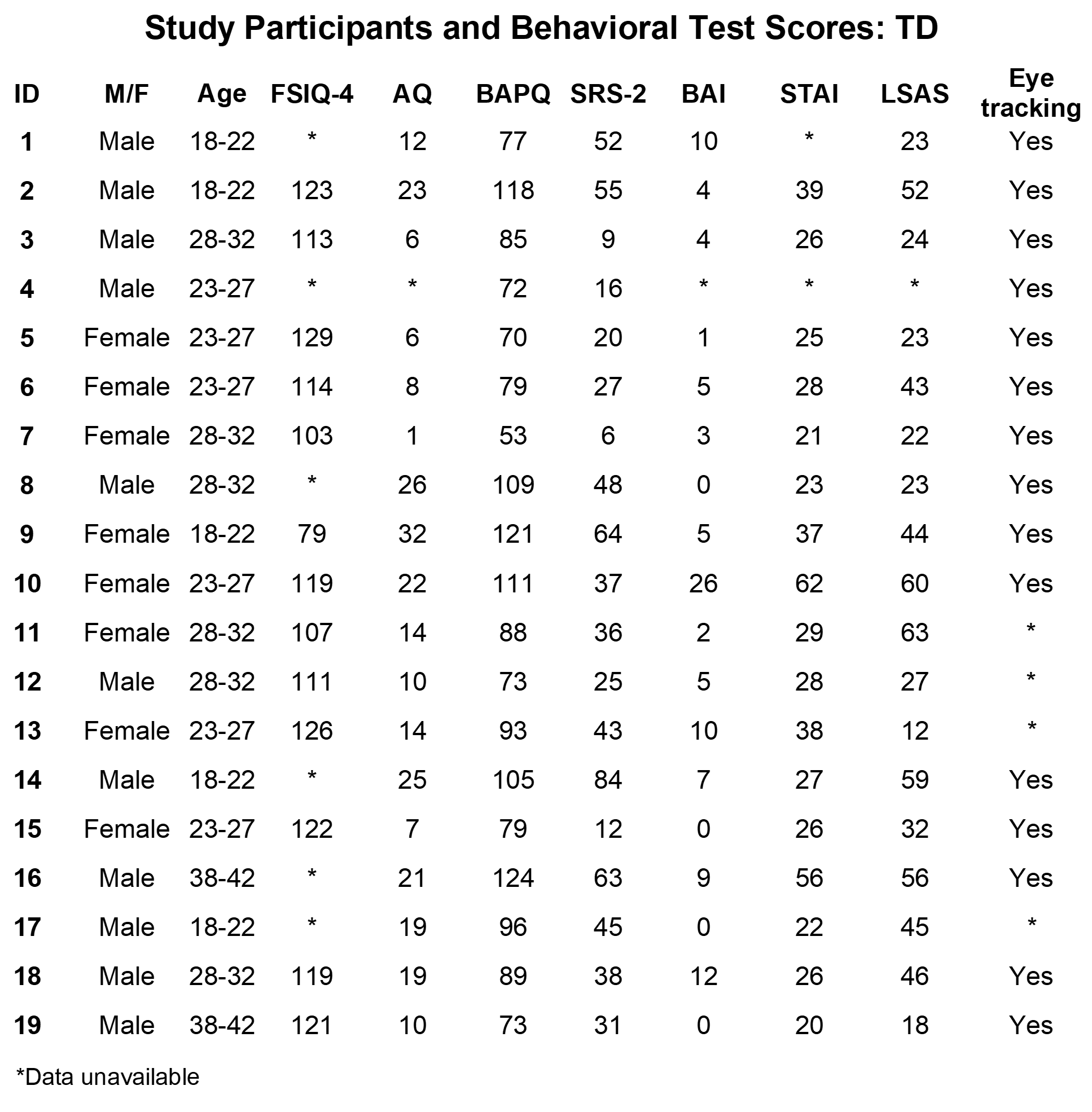

Supplement: S2 Table — Assessment measures include the Autism-Spectrum Quotient test (AQ, total scores); Broad Autism Phenotype Questionnaire (BAPQ, total scores); Social Responsiveness Scale, Second Edition (SRS-2, raw scores); Beck Anxiety Inventory (BAI, total scores); State-Trait Anxiety Inventory (STAI; total state anxiety scores); and the Liebowitz Social Anxiety Scale (LSAS, total scores). The Wechsler Abbreviated Scale of Intelligence, 2nd Edition (WASI-II) was administered to estimate full-scale intelligence quotient scores based on four subtests (FSIQ-4). *Indicates data were not acquired. (TIF) [file pone.0265798.s004.TIF]

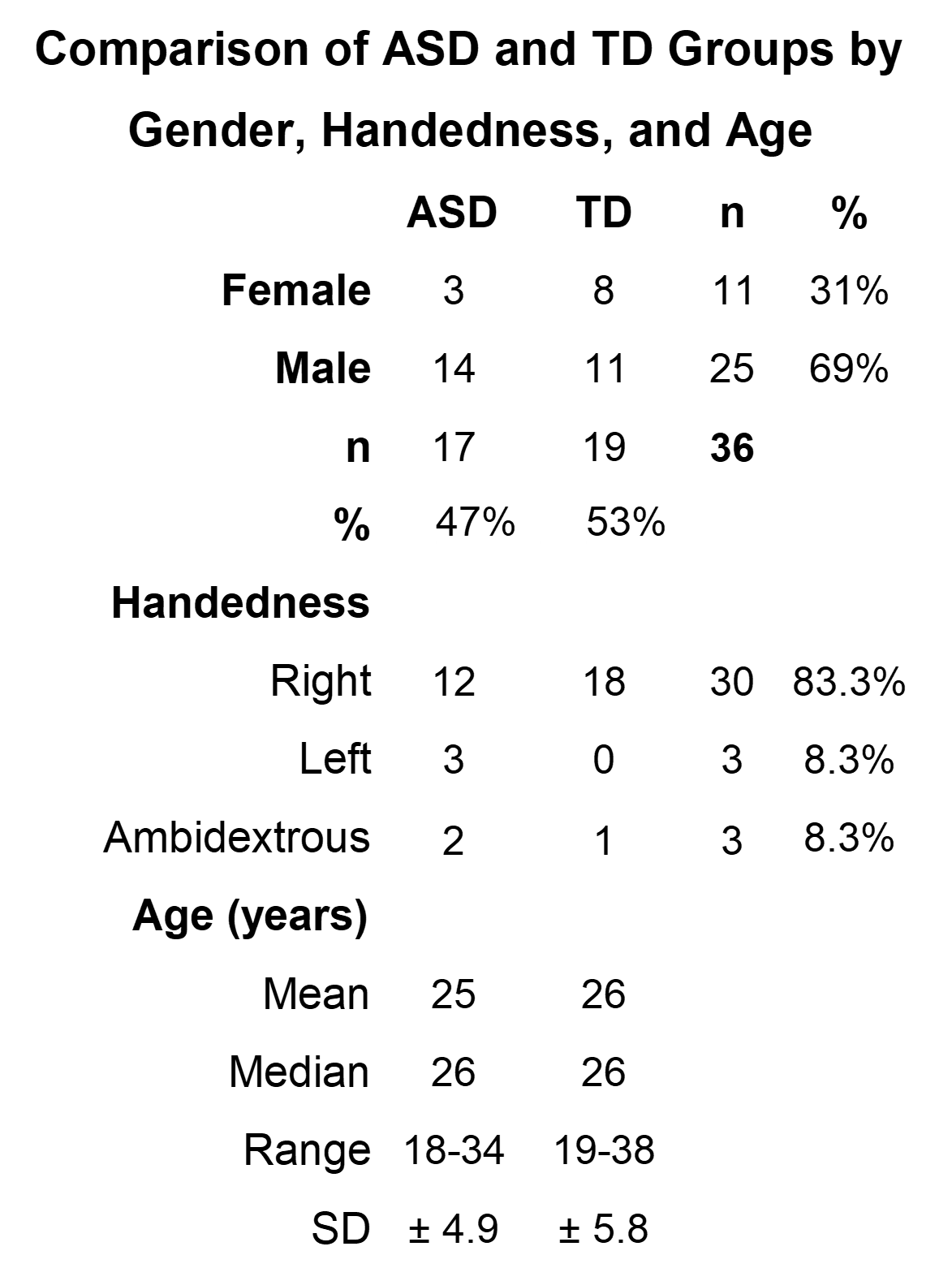

Supplement: S3 Table — Groups were similar in terms of age and handedness; however, the ratio of male to female participants was higher in the ASD group than in the TD group. The gender composition of the ASD group is consistent with the estimated 4:1 male:female ratio of ASD diagnosis. This ratio increases to 6 males diagnosed with ASD for every 1 female in people whose cognitive functioning is within or above normal limits, such as those in our sample (Kirkovski, M., Enticott, P. G., & Fitzgerald, P. B. (2013). A review of the role of female gender in autism spectrum disorders. Journal of Autism and Developmental Disorders, 43(11), 2584–2603). (TIF) [file pone.0265798.s005.TIF]

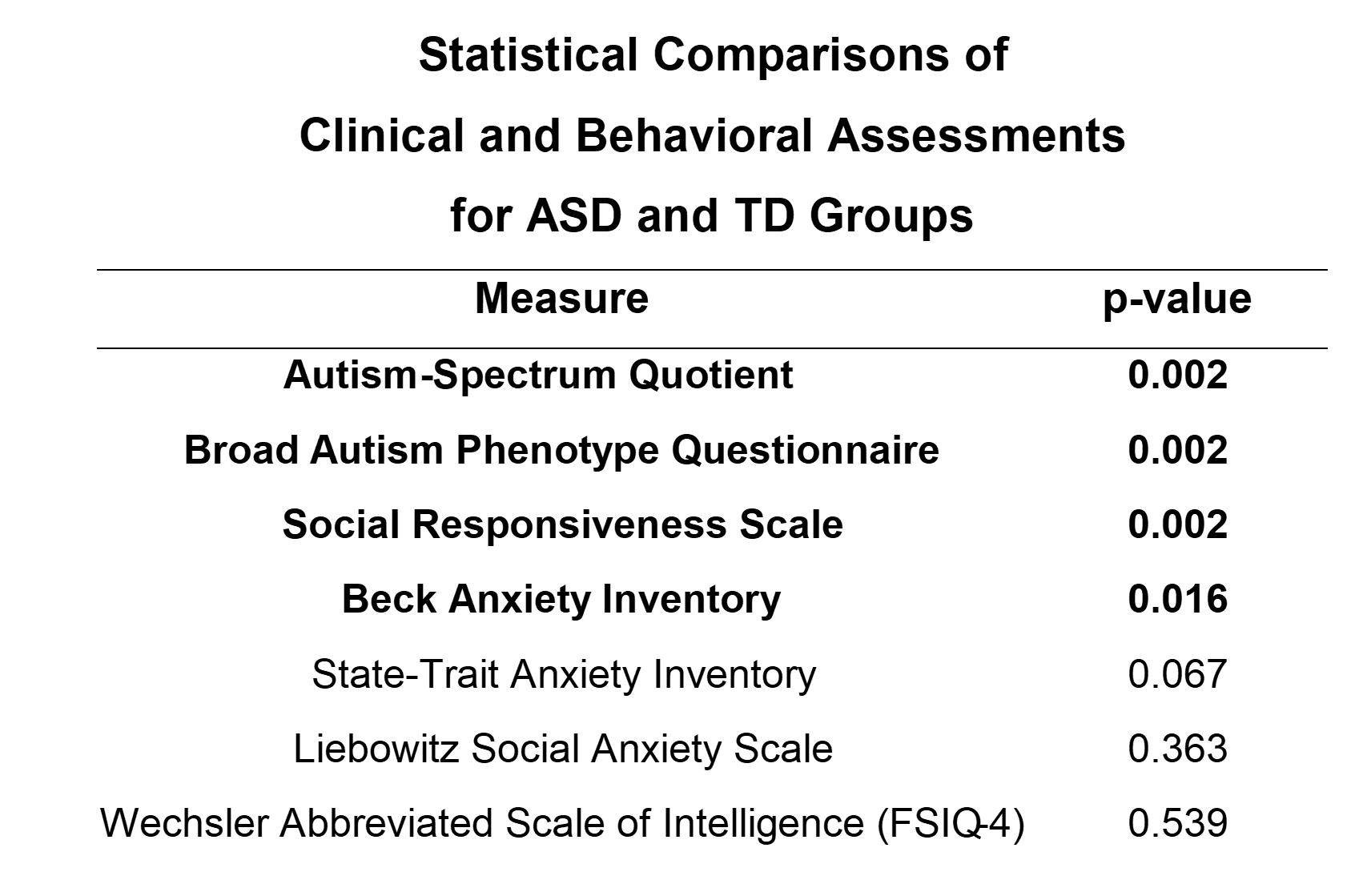

Supplement: S4 Table — No evidence was found for differences between the groups for FSIQ-4 (estimated by the Wechsler Abbreviated Scale of Intelligence); State-Trait Anxiety Inventory (state anxiety items only); or the Liebowitz Social Anxiety Scale, and is taken as evidence in favor of matched groups with respect to these metrics. (TIF) [file pone.0265798.s006.TIF]

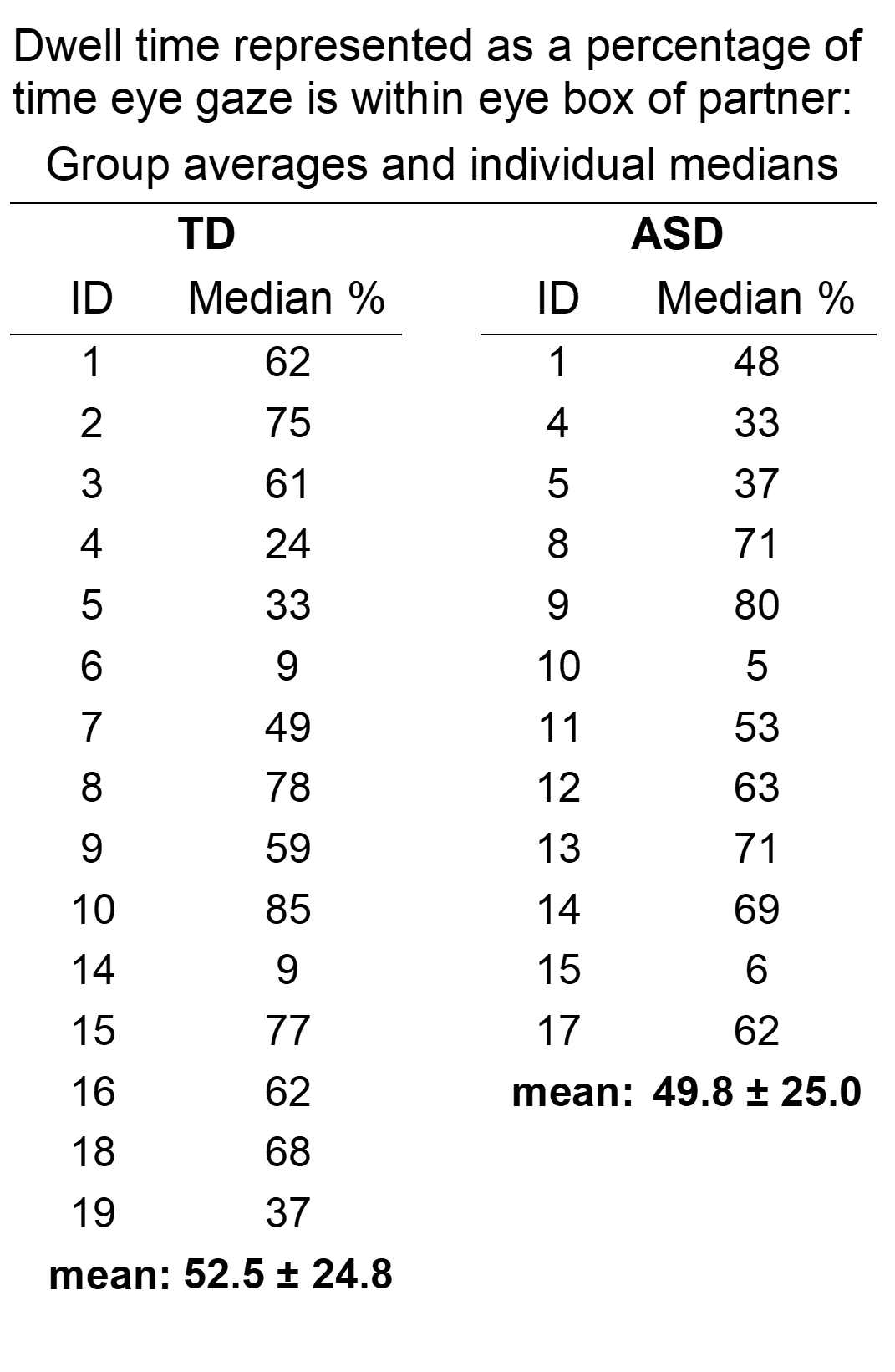

Supplement: S5 Table — Group averages and individual median percentages of eye-gaze time within the eye box of partners for typically developed (TD) participants (left column) and participants with autism spectrum disorder (ASD) (right column) during the Real Eye Condition. A t-test of these median percentages shows t(25) = 0.28 n.s. See S1 and S2 Figs for a graphical run-by-run representation of eye tracking performance. (TIF) [file pone.0265798.s007.TIF]
